# Association with the Quality of Sleep and the Mediating Role of Eating on Self-Esteem in Healthcare Personnel

**DOI:** 10.3390/nu11020321

**Published:** 2019-02-02

**Authors:** María del Carmen Pérez-Fuentes, María del Mar Molero Jurado, Ana Belén Barragán Martín, África Martos Martínez, José Jesús Gázquez Linares

**Affiliations:** 1 Department of Psychology, University of Almería, 04120 Almería, Spain; mmj130@ual.es (M.d.M.M.J.); abm410@ual.es (A.B.B.M.); amm521@ual.es (Á.M.M.); jlinares@ual.es (J.J.G.L.); 2 Department of Psychology, Universidad Autónoma de Chile, Santiago 4780000, Chile

**Keywords:** self-esteem, quality of sleep, eating, nursing

## Abstract

In recent decades, organizational research has paid special attention to the mechanisms promoting the health and well-being of nursing professionals. In this context, self-esteem is a personal resource associated with well-being at work and the psychological well-being of nurses. The purpose of this study was to analyze the mediating role of eating on the relationship between sleep quality and self-esteem in nursing professionals. A sample of 1073 nurses was administered the Rosenberg General Self-Esteem Scale, the Pittsburgh Sleep Quality Index (PSQI), and the Three-Factor Eating Questionnaire-R18 (TFEQ-18). The results show that poor sleep quality and type of eating directly and indirectly affect self-esteem. Poor sleep quality lowered self-esteem through emotional eating and, even though emotional eating facilitated uncontrolled eating, this relationship had no significant effect on self-esteem. The findings of this study suggest that hospital management should implement employee health awareness programs on the importance of healthy sleep and design educational interventions for improving diet quality.

## 1. Introduction

Positive Occupational Health Psychology (POHP) is a discipline for the “scientific study of optimal functioning of the health of persons and groups in organizations, the effective management of their psychosocial wellbeing at work, and the development of healthy organizations” [1] (p. 23). Far from the tendencies of more traditional research to concentrate on the negative aspects of health, this new vein of positive psychology emerged from the need to provide a better understanding of the mechanisms that promote the occupational health and well-being of workers [2,3].

One of the most influential theoretical frameworks in research on well-being and job stress is the Job Demands-Resources Model (JD-R) [4]. This model underlines the relevance of workers’ personal resources that contribute to their capacity for buffering the negative impacts of job demands, promoting job commitment and positively influencing job performance [5,6]. Personal resources can also drive growth and professional development and they are determining factors for workers’ psychological and occupational well-being [7]. In this context, self-esteem has been one of the personal resources that is most widely studied in the area of organization, although its value has also been widely recognized in education [8,9,10].

Self-esteem is a multidimensional construct referring to an individual’s evaluation of their own worth [11]. It determines the way we act and our social behavior, its relevance stemming from the effect that it has on individuals and different outcomes in their lives [12]. For example, high levels of self-esteem are related to higher satisfaction in interpersonal relations and at work [13], physical and psychological well-being [14], academic performance [15], and the effective management of stress and adequate coping in conflict situations [16]. Their level of self-esteem determines the success and well-being of people in important areas of life, such as health, social relations, work, and education [14,17].

In relation to organizations, empirical research in self-esteem has given special attention to nursing professionals who form the majority group in the healthcare profession [18]. Another reason for this interest is the nature of their work and the impact on their well-being. These include: (a) many of the tasks involved in the integral care of patients require demanding levels of physical activity (physical exhaustion); (b) permanent contact with patients and their families, involving continual exposure to suffering and illness (emotional and psychological burnout); and (c) attention to a high volume of patients during their work day (work overload) [19,20,21]. Thus workers’ positive personal evaluations increase their well-being and satisfaction, as well as improve the therapeutic relationship with their patients [22,23].

One question related to the health and satisfaction of nursing professionals addresses inadequate sleep, which could result from factors such as working rotating shifts and high levels of stress [24,25]. A sleep deficit, whether in amount or duration, negatively affects an individual’s physical and psychological health, socioemotional functioning, and psychosocial adjustment [26,27]. Because it reduces the tendency to think positively, it is linked with negative emotional states, reducing the motivation for gratifying social activities and adversely impacting interpersonal relations [28,29]. Insufficient sleep has also been associated with ineffective decision-making, stronger emotional reactions (e.g., irritability), an impulse control deficit, and a deterioration of emotional regulation—the main mechanism linking lack of sleep with psychological health [30,31]. However, poor quality sleep and short sleep duration in nursing professionals not only have negative consequences for them, but also for the organization. It diminishes job performance and service quality, increasing the risk of making mistakes on the job, and endangering patient safety [32].

In addition to the above, poor quality or insufficient sleep as a result of rotating shifts and job stress is in itself a factor that acts as an indicator of psychological stress. It can also lead to maladjustments in diet and eating behavior [25,33]. In a systematic review, it was observed that negative emotions can cause a feeling of being full, which leads to a decrease in the amount of food eaten. Conversely, the amount eaten may increase to alleviate the distress of negative emotions. This phenomenon has been explained as “emotional eating” [34]. For example, Van Strien and Koenders (2014) showed that an association between a short duration of sleep and an increase in body mass index (BMI) in women was due to emotional eating, and not from uncontrolled eating or cognitive restriction. Dweck et al. [33] found the same relationship between sleep quality and emotional and uncontrolled eating. Emotional eating has been considered a predictor of Binge Eating Disorder [35]. Similarly, it has been demonstrated in normal and obese populations that emotional eating is associated with a higher BMI, which may be reflected in weight gain [34]. Frederick, Sandhu, Morse, and Swami [36] showed that BMI is an important predictor of body image, where an increase in weight causes greater body dissatisfaction. Some authors, such as Griffiths et al. [37] and Oh, Song, and Shin [38], found that body dissatisfaction has adverse consequences on the emotional well-being of individuals, negatively affecting their self-esteem and the quality of their psychosocial life.

Because of the important implications that self-esteem, sleep, and eating behavior have for the general well-being of workers, our objective was to analyze the mediating role of eating on the impact that quality of sleep has on the level of self-esteem in nursing professionals.

The following hypotheses are proposed: (1) poor quality of sleep is negatively associated with emotional intake and uncontrolled intake; and (2) the self-esteem of health professionals is negatively associated with poor quality of sleep and the tendency towards emotional intake.

## 2. Materials and Methods

### 2.1. Participants

The original sample consisted of 1094 nurses in Andalusia (Spain). Incomplete questionnaires or those answered at random were discarded. The final study sample was made up of a total of 1073 Spanish nurses aged 22 to 57 years, with a mean age of 32.32 years (SD = 6.62). Of this sample, 14.7% (*n* = 158) were men and 85.3% (*n* = 915) women, with mean ages of 32.79 (SD = 6.27) and 32.24 years (SD = 6.68), respectively. The sample was distributed by sleep quality as follows: 60% (*n* = 644) had sleep problems and the remaining 40% (*n* = 429) had no sleep problems.

### 2.2. Instruments

Rosenberg Self-Esteem Scale [11]. Developed for evaluating self-esteem in adolescents, this scale consists of 10 items that focus on one’s feelings of respect and acceptance. The items are rated on a four-point Likert-type scale (from 1 = strongly agree, to 4 = strongly disagree). Other studies have demonstrated its adequate psychometric characteristics in both the general population [39] and in more specific populations [40]. In this study, the internal consistency was α = 0.82.

Pittsburgh Sleep Quality Index (PSQI) [41]; Spanish version by Macías and Royuela [42]. This questionnaire, which was developed to measure sleep quality, discriminates between good and poor sleepers. It consists of 24 items, with five for evaluation by a roommate or bed partner, which are not included in the subject’s self-evaluation score. The 19 self-reported items focus on aspects such as sleep latency and duration, frequency, and the severity of sleep problems. Seven components are generated based on the subject’s answers: subjective quality, latency, habitual sleep efficiency, disturbances, the use of sleeping medication, and repercussions on daytime activity. An overall sleep quality score was found from the sum of these partial components. A global score on the PSQI was taken for sleep quality, over a range of 0 to 21 points (0 points = no sleep problems and 21 points = the existence of severe problems in all areas or dimensions, as evaluated by the instrument). Royuela and Macías [43] reported reliability indices for the instrument of 0.81 in a clinical population and 0.67 in a sample of students.

Three-Factor Eating Questionnaire-R18 (TFEQ-R18). The brief version of the original 51-item TFEQ [44], translated and adapted to Spanish (TFEQ-SP) by Jáuregui-Lobera, García-Cruz, Carbonero-Carreño, Magallares, and Ruiz-Prieto [45] and adapted by Pérez-Fuentes, Molero, Gázquez, and Oropesa [46] for a nursing population, was used in this study. The questionnaire consists of 18 items rated on a four-point response scale (1 = definitely true, 2 = mostly true, 3 = mostly false, 4 = definitely false). It evaluates three dimensions of eating behavior: (a) uncontrolled eating (tendency to eat more than usual, due to a loss of control over eating, with a subjective sensation of hunger); (b) emotional eating (inability to resist emotional signals, eating as a response to negative emotions); and (c) cognitive restraint (conscious restraint of eating directed at controlling body weight and promoting weight loss). The TFEQ-R18 shows adequate coefficients of reliability on all three subscales (varying from 0.75 to 0.87) [45] and is adequate in the nursing population (0.85–0.90) [46]. In this study, the reliability indices were 0.89 for uncontrolled eating, 0.84 for emotional eating, and 0.74 for cognitive restriction. 

### 2.3. Procedure

Before collecting data, the following were guaranteed to participants: compliance with information standards, confidentiality, and ethics in data processing. All subjects gave their informed consent for inclusion prior to participating. The study was conducted in accordance with the Declaration of Helsinki and was approved by the Bioethics Committee at the University of Almería. The questionnaires were administered on a web platform that enabled participants to complete them online. For the control of random or incongruent answers, a series of control questions were included and any such cases were discarded from the study sample.

### 2.4. Data Analysis

To test the relationship between the variables included in the causal analyses, bivariate correlations were calculated. Then the descriptive statistics for these variables were found. To test for the existence of significant differences in self-esteem and eating between the groups with and without sleep problems, a Student’s *t*-test for independent samples was conducted. To classify the groups according to the quality of sleep, we followed the proposal of Buysse et al. [41], where a Global PSQI >5 suggests severe problems in sleep in at least two areas, or moderate problems in more than three areas.

The Preacher and Hayes [47] macro for SPSS was used to estimate the mediation model, in this case for multiple mediation [48]. This resource enables the computation of different regression models, finding information on indirect mediation and avoiding the limitations of the classical Baron and Kenny [49] proposal. To do this, bootstrapping was applied with 5000 bootstraps, which provided a confidence interval of 95% and determined the multiple mediation of the mediator variables. In this study, an analysis of multiple mediation was carried out with two mediator variables forming a causal chain.

## 3. Results

### 3.1. Descriptive and Correlational Analyses

Table 1 shows the descriptive statistics and correlations between variables: overall self-esteem, sleep quality, and eating.

The table shows data confirming the existence of a negative correlation (*r* = −0.23, *p* < 0.001) between the predictor variable (global PSQI score) and self-esteem as the dependent variable. Furthermore, of the variables considered as potential mediators (uncontrolled eating, emotional eating, and cognitive restraint), those observed to maintain correlations, in this case negative, with the dependent variable, were uncontrolled eating (*r* = −0.21, *p* < 0.001) and emotional eating (*r* = −0.24, *p* < 0.001). Therefore, these are the variables that were included later as mediators in the analysis.

Table 2 shows the results of the analysis of the mean scores in self-esteem and the TFEQ-R18 subscales for a comparison of subjects with and without sleep problems. The results reveal the existence of significant differences in relation to the level of self-esteem, (*t*_(1071)_ = 5.01; *p* < 0.001; *d* = 0.031) between those who had sleep problems (*M* = 31.88; *SD* = 4.73) and those who did not (*M* = 33.25; *SD* = 4.12), where the latter had higher scores.

In addition, when the groups were compared for the eating dimensions that correlated with sleep quality, statistically significant differences were observed in uncontrolled eating (*t*_(1071)_ = −4.96; *p* < 0.001; *d* = 0.31) and emotional eating (*t*_(1071)_ = −4.21; *p* < 0.001; *d* = 0.26), where those who had sleep problems had the highest points in both cases. There were no statistically significant differences between groups in cognitive restraint (*t*_(1071)_ = −1.80; *p* = 0.071).

### 3.2. Multiple Mediation Analysis

The mediation analysis was carried out based on the following mediation hypothesis: Having sleep problems involves a tendency towards emotional eating (E-E), which has a negative repercussion on self-esteem. Emotional eating facilitates uncontrolled eating (U-E), although it does not have indirect mediations on self-esteem by this path.

For the computation of the model, the PSQI global score was taken as the independent or predictor variable. In this case, the variable was previously dichotomized following the authors’ [41] proposal. We therefore had two groups coded as 0 = no sleep problems and 1 = sleep problems. The dependent variable proposed in the model was self-esteem and, as mediators, emotional eating (M_1_) and uncontrolled eating (M_2_).

**Figure 1:**
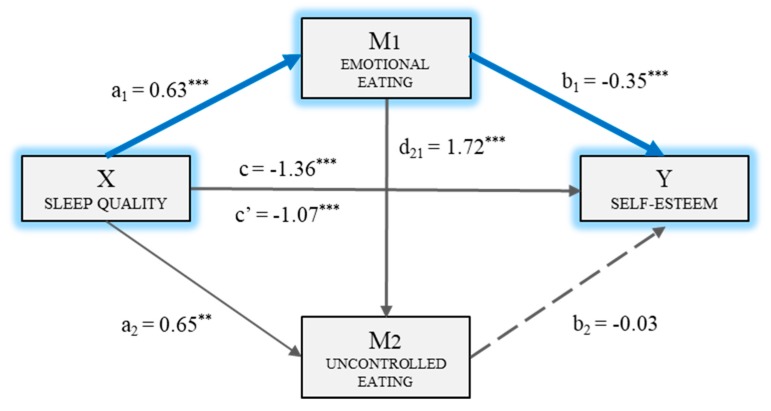
Multiple mediation model of eating (emotional/uncontrolled) on the relationship between sleep quality and self-esteem. ** *p* < 0.01, *** *p* < 0.001.

Thus, the multiple mediation model was computed with two mediating variables (M_1_: E-E and M_2_: U-E). Figure 1 shows the model and includes the direct, indirect, and total mediations. Firstly, it may be observed that there was a statistically significant mediation (*B*_PSQI_ = 0.63, *p* < 0.001) of sleep quality (X) on emotional eating (M_1_). The second regression analysis took Mediator 2 as the result variable, and included sleep quality (X) and emotional eating (M_1_) in the equation. There was a significant mediation of emotional eating (*B*_E-E_ = 1.72, *p* < 0.001) and of sleep quality (*B*_PSQI_ = 0.65, *p* < 0.01) on uncontrolled eating (M_2_). The third regression analysis, taking self-esteem (Y) as the result variable, estimated the mediation of the independent variable, and of the two mediators. In this case, the mediations of emotional eating (*B*_E-E_ = −0.35, *p* < 0.001) and sleep quality (*B*_PSQI_ = −1.07, *p* < 0.001) as the independent variable were significant. Meanwhile, uncontrolled eating (M_2_) did not have a significant mediation (*B*_U-E_ = −0.03 *p* = 0.265) on the dependent variable. The total mediation of sleep quality on self-esteem was significant (*B*_PSQI_ = −1.36, *p* < 0.001).

Finally, the analysis of the indirect mediation by bootstrapping found that the resulting data supported the significance of Path 1 (Ind_1_: sleep quality→ emotional eating → self-esteem; *B* = −0.22, *SE* = 0.07, 95% *CI* (−0.41, −0.10)). Therefore, sleep quality had a stronger mediation on self-esteem through emotional eating (M_1_) than the two mediators operating in series. Thus, the path or indirect mediation would basically take place through emotional eating.

## 4. Discussion

Most nursing professionals showed poor sleep quality (60%), which could be the result of working in rotating shifts and high job stress [24,25]. The results also showed that poor sleep quality negatively related to self-esteem, suggesting that inadequate sleep reduces the tendency to think positively and is related to negative emotional states that affect the emotional and psychological well-being of nurses [28].

The data show that poor sleep quality functions as a predictor of eating behavior. Specifically, workers with poorer sleep quality had higher rates of emotional and uncontrolled eating. Similarly, Dweck et al. [33] suggested that both eating styles are associated with poor sleep quality, and not its duration. This finding could be explained by a deficit of impulse control and a deterioration of emotional regulation and decision-making caused by deficient sleep quality [29]. In agreement with previous studies, no significant relationship was found between sleep quality and cognitive restriction [10].

The mediation models confirmed our hypotheses. In the first place, a deficit in sleep quality implies more emotional eating, which negatively affects self-esteem. Poor sleep quality causes generalized emotional distress. In response to those negative emotional signals, people tend to eat more to feel better. Some authors have noted that this is an atypical response of the organism, which may be explained by inadequate emotional regulation. It has also been suggested that emotional eating is related to maladaptive coping strategies [50]. In addition, these sleep problems are associated with eating more junk foods, which are high in fats and sugars, therefore emotional eating leads to weight gain [27,34]. Thus, individuals faced with weight gain feel greater dissatisfaction with their body image and this negatively affects their self-esteem and their psychosocial quality of life [36,38]. As self-esteem is a personal resource determining workers’ psychological well-being and their well-being at work, lower self-esteem will impact their job performance and the quality of service to patients will be reduced [5,22].

Emotional eating facilitates uncontrolled eating, although it does not affect self-esteem, even though these two different dimensions of eating behavior are closely related. Emotional eating is provoked by an absence of adapted emotional regulation. This is associated with a deficit in impulse control, which is more characteristic of uncontrolled eating [29,35]. The findings of Van Strien and Koenders [50] offer a possible explanation for the absence of an association between uncontrolled eating and self-esteem. These authors showed that self-esteem is only weakened when there is an increase in BMI. However, this relationship was only found when emotional eating was the only mediator between poor sleep quality and self-esteem.

The results of this study have relevant practical implications. For one thing, the importance of self-esteem as an essential personal resource for nursing professionals [7,8] should be emphasized. For another, the relationship of sleep quality and diet on the general well-being of nurses and the quality of their attention to patients should also be underlined [28,34]. Organizations should implement health awareness programs for workers emphasizing the importance of quality sleep to prevent health problems, as well as educational programs to facilitate tools that improve the quality of their diet [32]. Following the recommendations of Amutio et al. [26], it would also be of interest to include positive interventions (e.g., mindfulness) to improve the quality of sleep of these healthcare professionals. 

This study has some limitations. First, its cross-sectional nature impedes the establishment of any causal relationship between the study variables, for which a longitudinal design would be necessary. The second is that the data may be biased by the variance in the common method, because of how the data were collected. Therefore, we propose the inclusion of other qualitative methods and more objective and exhaustive measures to offer more precise information on the duration and quality of sleep. Finally, the sample is made up of a majority of women, making it difficult to generalize the results to the entire group.

For future lines of research it is suggested that variables that are related to work demands (e.g., shifts) be included with individual and collective impacts on the organization (e.g., engagement, job performance). This study did not measure this factor, which could had been a confounder. Similarly, multilevel studies of the deficit in sleep quality in different areas of work would be of interest for implementing preventive programs.

## 5. Conclusions

This study analyzed the mediating role of eating on the association of sleep quality with self-esteem in nursing professionals. It emphasizes the importance of self-esteem in the organizational environment as a personal resource that is essential to the psychological and emotional well-being of workers, in addition to positively influencing organizational results (e.g., job performance) and improving therapeutic relations with patients.

Furthermore, the TFEQ-R18 [46] is a valid instrument for studying the three dimensions of eating behavior (emotional, behavioral, and cognitive)—not only for persons with obesity, but also in the normal population. 

This study provides important contributions to Positive Occupational Health Psychology (POHP) by emphasizing eating behavior and sleep quality as essential aspects for the health and well-being of nursing professionals and the quality of patient care. It was demonstrated that both factors have a direct and indirect relationship with self-esteem.

The main finding of this study is that poor sleep quality can lower the self-esteem of nurses through emotional eating, suggesting that this relationship could cause job performance to diminish and patient service quality to deteriorate. The second most important finding is that there is a close relationship between the emotional dimension of eating (emotional eating) and its behavioral dimension (uncontrolled eating). However, this association does not negatively affect the self-esteem of nursing professionals.

The results suggest the relevance of implementing health awareness programs to make healthcare professionals aware of the importance of healthy sleep and educational programs for improving the quality of their diet. However, it is necessary to include other variables of interest (e.g., shift work, eating habits, or BMI in health professionals), which should be involved in the proposed model.

## Figures and Tables

**Table 1:** Descriptive statistics and correlations between self-esteem, sleep quality, and eating variables.

	*M*	*SD*	(1)	(2)	(3)	(4)	(5)
(1) Self-esteem	32.43	4.54					
(2) Sleep quality	6.44	2.90	−0.23 ***				
(3) Uncontrolled eating	17.38	5.88	−0.21 ***	0.17 ***			
(4) Emotional eating	5.75	2.50	−0.24 ***	0.19 ***	0.74 ***		
(5) Cognitive restraint	16.08	4.55	−0.002	0.05	0.23 ***	0.25 ***	

*** *p* < 0.001.

**Table 2:** Self-esteem and eating (emotional/uncontrolled). Descriptive statistics and *t*-test by sleep quality (no problems/problems).

	Sleep Quality						*t*	*p*	*d*
	No Problems			Problems					
	*N*	*Mean*	*SD*	*N*	*Mean*	*SD*			
Self-esteem	429	33.25	4.12	644	31.88	4.73	5.01 ***	0.000	0.31
Uncontrolled eating	429	16.32	5.36	644	18.08	6.11	−4.96 ***	0.000	0.31
Emotional eating	429	5.37	2.28	644	6.01	2.60	−4.21 ***	0.000	0.26
Cognitive restraint	429	15.77	4.53	644	16.28	4.55	−1.80	0.071	--

*** *p* < 0.001.

## References

[1] Salanova M., Martínez I.M., Llorens S. (2014). A more “positive “look at occupational health from positive organizational psychology during crisis times: Contributions from the WoNT research team. Pap. Psicól..

[2] Bakker A.B., Rodríguez-Muñoz A. (2012). Introducción a la psicología de la salud ocupacional positiva. Psicothema.

[3] Bakker A.B., Rodríguez-Muñoz A., Derks D. (2012). La emergencia de la psicología de la salud ocupacional positiva. Psicothema.

[4] Demerouti E., Bakker A.B., Nachreiner F., Schaufeli W.B. (2001). The job demands resources model of burnout. J. Appl. Psychol..

[5] Bakker A.B., Demerouti E. (2017). Job demands-resources theory: Taking stock and looking forward. J. Occup. Health Psychol..

[6] Lisbona A., Palaci F., Salanova M., Frese M. (2018). The effects of work engagement and self-efficacy on personal initiative and performance. Psicothema.

[7] Xanthopoulou D., Bakker A.B., Demerouti E., Schaufeli W.B. (2009). Reciprocal relationships between job resources, personal resources, and work engagement. J. Vocat. Behav..

[8] Molero M.M., Pérez-Fuentes M.C., Gázquez J.J., Barragán A.B. (2018). Burnout in Health Professionals According to Their Self-Esteem, Social Support and Empathy Profile. Front. Psychol..

[9] Pérez-Fuentes M.C., Gázquez J.J. (2010). Variables relacionadas con la conducta violenta en la escuela según los estudiantes. Rev. Int. Psicol. Ter. Psicol..

[10] Van Wingerden J., Derks D., Bakker A.B. (2017). The impact of personal resources and job crafting interventions on work engagement and performance. Hum. Resour. Manag..

[11] Rosenberg M. (1965). Society and the Adolescent Self-Image.

[12] Simón M.M., Molero M.M., Pérez-Fuentes M.C., Gázquez J.J., Barragán A.B., Martos Á. (2017). Análisis de la relación existente entre el apoyo social percibido, la autoestima global y la autoeficacia general. Eur. J. Health Res..

[13] Orth U., Robins R.W., Widaman K.F. (2012). Life-span development of self-esteem and its effects on important life outcomes. J. Pers. Soc. Psychol..

[14] Orth U., Specht J. (2017). The lifespan development of self-esteem. Personality Development Across the Lifespan.

[15] Pérez-Fuentes M.C., Molero M.M., Barragán A.B., Martos A., Simón M.M., Gázquez J.J. (2018). Self-efficacy and engagement in health science students and their relation to self-esteem [Autoeficacia y Engagement en estudiantes de Ciencias de la Salud y su relación con la autoestima]. Publicaciones.

[16] Yildirim N., Karaca A., Cangur S., Acikgoz F., Akkus D. (2017). The relationship between educational stress, stress coping, self-esteem, social support, and health status among nursing students in Turkey: A structural equation modeling approach. Nurs. Educ. Today.

[17] Orth U., Robins R.W. (2014). The development of self-esteem. Curr. Dir. Psychol. Sci..

[18] World Health Organization (2016). World Health Statistics 2016: Monitoring Health for the SDGs Sustainable Development Goals.

[19] Adriaenssens J., De Gucht V., Maes S. (2015). Determinants and prevalence of burnout in emergency nurses: A systematic review of 25 years of research. Int. J. Nurs. Stud..

[20] Cooper S.L., Carleton H.L., Chamberlain S.A., Cummings G.G., Bambrick W., Estabrooks C.A. (2016). Burnout in the nursing home health care aide: A systematic review. Burnout Res..

[21] Banerjee S.C., Manna R., Coyle N., Shen M.J., Pehrson C., Zaider T., Hammonds S., Krueger C.A., Parker P.A., Bylund C.L. (2016). Oncology nurses’ communication challenges with patients and families: A qualitative study. Nurse Educ. Pract..

[22] Edwards D., Burnard P., Bennett K., Hebden U. (2010). A longitudinal study of stress and self-esteem in student nurses. Nurs. Educ. Today.

[23] Goñi E., Esnaola I., Rodríguez-Fernández A., Camino I. (2015). Personal self-concept and satisfaction with life in adolescence, youth and adulthood. Psicothema.

[24] Chappel S.E., Verswijveren S.J., Aisbett B., Considine J., Ridgers N.D. (2017). Nurses’ occupational physical activity levels: A systematic review. Int. J. Nurs. Stud..

[25] Li J., Pursey K., Duncan M., Burrows T. (2018). Addictive Eating and Its Relation to Physical Activity and Sleep Behavior. Nutrients.

[26] Amutio A., Franco C., Sánchez-Sánchez L.C., Pérez-Fuentes M.C., Gázquez-Linares J.J., van Gordon W., Molero-Jurado M.M. (2018). Effects of mindfulness training on sleep problems in patients with fibromyalgia. Front. Psychol..

[27] Sun Q., Ji X., Zhou W., Liu J.D. (2018). Sleep problems in shift nurses: A brief review and recommendations at both individual and institutional levels. J. Nurs. Manag..

[28] Becker N.B., de Jesus S.N., Viseu J.N., Stobäus C.D., Guerreiro M., Domingues R.B. (2018). Depression and quality of life in older adults: Mediation effect of sleep quality. Int. J. Clin. Health Psychol..

[29] Palmer C.A., Alfano C.A. (2017). Sleep and emotion regulation: An organizing, integrative review. Sleep Med. Rev..

[30] Beattie L., Kyle S.D., Espie C.A., Biello S.M. (2015). Social interactions, emotion and sleep: A systematic review and research agenda. Sleep Med. Rev..

[31] Tempesta D., Socci V., De Gennaro L., Ferrara M. (2017). Sleep and emotional processing. Sleep Med. Rev..

[32] Beebe D., Chang J.J., Kress K., Mattfeldt-Beman M. (2017). Diet quality and sleep quality among day and night shift nurses. J. Nurs. Manag..

[33] Dweck J.S., Jenkins S.M., Nolan L.J. (2014). The role of emotional eating and stress in the influence of short sleep on food consumption. Appetite.

[34] Devonport T.J., Nicholls W., Fullerton C. (2017). A systematic review of the association between emotions and eating behaviour in normal and overweight adult populations. J. Health Psychol..

[35] Escandón-Nagel N., Peró M., Grau A., Soriano J., Feixas G. (2018). Emotional eating and cognitive conflicts as predictors of binge eating disorder in patients with obesity. Int. J. Clin. Health Psychol..

[36] Frederick D.A., Sandhu G., Morse P.J., Swami V. (2016). Correlates of appearance and weight satisfaction in a US national sample: Personality, attachment style, television viewing, self-esteem, and life satisfaction. Body Image.

[37] Griffiths S., Murray S.B., Bentley C., Gratwick-Sarll K., Harrison C., Mond J.M. (2017). Sex differences in quality of life impairment associated with body dissatisfaction in adolescents. J. Adolesc. Health.

[38] Oh E., Song E., Shin J. (2017). Individual Factors Affecting Self-esteem, and Relationships Among Self-esteem, Body Mass Index, and Body Image in Patients with Schizophrenia. Arch. Psychiatr. Nurs..

[39] Atienza F.L., Balaguer I., Moreno Y. (2000). Análisis de la dimensionalidad de la escala de autoestima de Rosenberg en una muestra de adolescentes valencianos. Rev. Psicol. Univ. Tarracon..

[40] Vázquez A.J., Vázquez-Morejón R., Bellido G. (2013). Fiabilidad y validez de la Escala de Autoestima de Rosenberg (EAR) en pacientes con diagnóstico de psicosis. Apuntes Psicol..

[41] Buysse D.J., Reynolds C.F., Monk T.H., Berman S.R., Kupfer D.J. (1989). The Pittsburgh Sleep Quality Index: A new instrument for psychiatric practice and research. Psychiatr. Res..

[42] Macías J.A., Royuela A. (1996). La versión española del índice de calidad de sueño de Pittsburgh. Inf. Psiquiátr..

[43] Royuela A., Macías J.A. (1997). Propiedades clinimétricas de la versión castellana del Cuestionario de Pittsburgh. Vigilia-Sueño.

[44] Stunkard A.J., Messick S. (1985). The three-factor eating questionnaire to measure dietary restraint, disinhibition and hunger. J. Psychosom. Res..

[45] Jáuregui-Lobera I., García-Cruz P., Carbonero-Carreño R., Magallares A., Ruiz-Prieto L. (2014). Psychometric properties of Spanish version of the Three-Factor Eating Questionnaire-R18 (TFEQ-SP) and its relationship with some eating and body image-related variables. Nutrients.

[46] Pérez-Fuentes M.C., Molero M.M., Gázquez J.J., Oropesa N.F. (2019). Psychometric properties of the Three Factor Eating Questionnaire in healthcare personnel. Nutr. Hosp..

[47] Preacher K.J., Hayes A.F. (2004). SPSS and SAS Procedures for estimating indirect effects in simple mediation models. Behav. Res. Methods Instrum. Comput..

[48] Preacher K.J., Hayes A.F. (2008). Asymptotic and resampling strategies for assessing and comparing indirect effects in multiple mediator models. Behav. Res. Methods Instrum. Comput..

[49] Baron R.M., Kenny D.A. (1986). The moderator-mediator variable distinction in social psychological research: Conceptual, strategic, and statistical considerations. J. Pers. Soc. Psychol..

[50] Van Strien T., Koenders P.G. (2014). Effects of emotional eating and short sleep duration on weight gain in female employees. J. Occup. Environ. Med..

